# How much are we worth? Experiences of nursing assistants in Swedish nursing homes during the first wave of COVID‐19

**DOI:** 10.1111/opn.12498

**Published:** 2022-08-17

**Authors:** Monica Bergqvist, Pia Bastholm‐Rahmner, Lars L Gustafsson, Katarina Holmgren, Anikó Veg, Caroline Wachtler, Katharina Schmidt‐Mende

**Affiliations:** ^1^ Division of Nursing, Department of Neurobiology, Care Sciences and Society Karolinska Institutet Huddinge Sweden; ^2^ Division of Clinical Pharmacology, Department of Laboratory Medicine, Karolinska Institutet Karolinska University Hospital Stockholm Sweden; ^3^ Academic Primary Care Center Stockholm Sweden; ^4^ Health Care Administration Stockholm Sweden; ^5^ Division of Family Medicine and Primary Care, Department of Neurobiology, Care Sciences and Society Karolinska Institutet Huddinge Sweden

**Keywords:** COVID‐19, interprofessional care, leadership, management, nursing assistants, nursing home care, older people, qualitative research, teamwork

## Abstract

**Background:**

NHs have been severely exposed during the COVID‐19 pandemic. Little is known about how staff who provide practical daily care of older residents experienced work during the pandemic. The aim of this study was to understand how nursing assistants (NAs) experienced their work at nursing homes (NHs) for older people during the first wave of the COVID‐19 pandemic.

**Methods:**

We conducted a qualitative study of focus group discussions with in total 20 participants from four NHs in Stockholm, Sweden. Discussions were held in November 2020. Transcripts were analyzed using inductive thematic analysis.

**Results:**

We identified three major themes: 1) We felt abandoned, scared and disrespected, 2) We made sure we made it through, and 3) We can do good work with appropriate resources. NAs felt disregarded as they were often left alone without adequate support from managers, registered nurses and the municipalities. NAs felt distressed and guilty and developed their own strategies to cope and manage their work.

**Conclusion and Implication for Practice:**

During the first wave of the COVID‐19 pandemic NAs felt abandoned and burdened due to lack of leadership. Organizational improvements are required to protect the wellbeing of NAs and to ensure sustainability of patient safety. NAs are crucial in the care for vulnerable older people and their experiences should constitute a keystone for development of future policy and practice in NHs.

**What does this research add to existing knowledge in gerontology?**

- Our research contributes to knowledge about how nursing assistants experience their situation during the COVID‐19 pandemic. The findings may be used for future planning in nursing homes.
- Nursing assistants reported feeling abandoned and burdened when left without robust leadership and support during the first wave of the pandemic.
- In literature, there is an overall lack of research from the perspective of nursing assistants who work closer than any other occupational category to the older people in nursing homes.

**What are the implications of this new knowledge for nursing care with older people?**

- Nursing assistants have an essential role in providing hands‐on care for vulnerable older people. They should be integrated into interprofessional teams with robust leadership, to ensure safe and high‐quality care.
- Nursing assistants need the support of clear and structured guidelines to perform their work according to best practice.
- A combination of peer, organisational and professional support is essential to maintain patient safety and good work environment for front line staff in nursing homes.

**How could the findings be used to influence policy or practice or research or education?**

- Nursing assistants should be given the opportunity to share their experiences, which may increase their own as well as residents´ wellbeing.
- The experiences of nursing assistants should be actively included in future research and in the development of nursing home care and routines.
- More research is required to understand how to sustainably support nursing assistants' work.

## 1 INTRODUCTION

During Spring 2020, health and social care professionals across the world were faced by an unpredictable spread of COVID‐19. Especially staff in nursing homes (NHs), who care for vulnerable older people, were forced to work under extreme pressure both physically and emotionally (Liu et al., 2020; Shanafelt et al., 2020). Overnight, they had to adapt to new and inconvenient working procedures. In the first phase of the pandemic, many NHs lacked access to testing and appropriate personal protective equipment (PPE) (Hoernke et al., 2021; WHO, 2020). Furthermore, to prevent spread of infection among residents many NHs banned visitors, leading to social isolation of residents (Prins et al., 2021; Verbeek et al., 2020).

NH residents worldwide have been highly affected by COVID‐19 and have suffered the highest number of deaths globally (WHO, 2020). Older people living in NHs are similar across Europe. They suffer from frailty, multimorbidity and often dementia which requires complex care and interprofessional teamwork as well as integrated leadership (Szebehely, 2020). NHs are designed to resemble the home environment, not primarily to prevent spread of infections, making them vulnerable to viral spread (Crotty et al., 2020; White et al., 2021.) The COVID‐19 pandemic revealed vulnerabilities in NHs. In Sweden, the pandemic revealed shortcomings in NH care including structural problems, insufficient funding, strained working conditions, undervalued and poorly trained staff and poor integration of nursing and medical care (Szebehely, 2020; The Health and Social Care Inspectorate, 2021).

Frontline staff in NHs in different countries such as China, Italy, Mexico, Peru, Spain, United States and Sweden shared similar experiences of working under extreme pressure during the first wave of the pandemic (Ecker et al., 2021; Liu et al., 2020; Sarabia‐Cobo et al., 2021; Szebehely, 2020). NHs staff closest to residents are called practical nurses, care aides or nursing assistants in Sweden (hereafter referred to as nursing assistants, NAs) and have various levels of professional training, from no specific education up to 3 years of vocational training. NAs provide most hands‐on care including assistance with hygiene, meals and mobilisation. In the daily work, NAs in Sweden should be supervised and work in teams with registered nurses (RNs).

The scientific literature has had focus on registered nurses (RNs) and physicians' experiences during the pandemic (Digby et al., 2021; Galehdar et al., 2020; Nyashanu et al., 2020; Sarabia‐Cobo et al., 2021; Shanafelt et al., 2020). So far, there has been little research on how NAs experienced their work at NHs during the pandemic (Ecker et al., 2021). We set out to address this gap. With a qualitative approach, a deeper insight into the NAs´ experiences from the crisis can be provided.

### 1.1 Aim

The aim of this study is to understand NAs´ experiences of working in NHs in Sweden during the first wave of the COVID‐19 pandemic.

## 2 MATERIALS AND METHODS

### 2.1 Study design

This is a qualitative descriptive study, aiming for in‐depth understanding of NAs work experiences during the first wave of COVID‐19. We used focus group discussions (FGDs) as our data collection method. In a group discussion, participants help each other to remember, talk to each other, ask questions and comment on each other's experiences (Kitzinger). Compared with individual interviews, FGDs help participants not only to explore and clarify their views, but also to articulate them (Kitzinger, 1995). Furthermore, group interaction during a FGD may facilitate the discussion of sensitive topics (Kvale & Brinkmann, 2009). Data from the FGDs were analysed using inductive thematic analysis (Braun & Clarke, 2006). In this approach, the themes identified are strongly linked to the collected data without trying to fit them into a pre‐existing theoretical frame (Braun & Clarke, 2006; Patton, 1990).

### 2.2 Setting and participants

The study took place in Region Stockholm, Sweden, which has a total of 318 NHs. We identified 82 NHs with more than 70 residents to ensure that the NHs had enough staff to allow five to eight NAs to participate in an FGD. We informed managers of these NHs about the study aim by e‐mail and invited their participation. Answers were received from 22 municipality managed NHs, of which 18 declined and four accepted. Declines were due to shortages in time and staff associated with the pandemic. Included NHs were located in areas with varying socioeconomic status, and all were affected to some degree by the pandemic (Supplement 1).

The sampling criteria for recruiting NAs were: (1) NA had worked during outbreak of COVID‐19 in spring 2020 and (2) NA wanted to share their experiences. Each manager recruited five to seven NAs and forwarded the names of those interested to the research team. All interested NAs received information about the study aim and that participation was voluntary and confidential. Twenty‐two NAs expressed interest, but due to sickness (2 NAs) the final number of participants was 20 (19 women, one man). Participants average age were between 51 and 60 years and most of them had more than 5 years of experience working in the NH (Supplement 1). All NAs were permanently employed.

### 2.3 Data collection

We conducted four FGDs (one at each of the selected NHs) between 11 November and 27 November, 2020. Each FGD included between three and seven participants. Two experienced researchers took part, one who moderated the discussions and one who took notes for follow‐up questions.

To ensure that different aspects of NAs' experience were elicited, we followed an interview guide (Box 1). The guide was pilot tested in an interview with one RN and one NA and refined before being used in the study. NAs were encouraged to talk about their experiences from the start of the pandemic in March 2020 to the FGD date, that is before the second pandemic wave in Sweden. FGDs lasted between one and a half to 2 h and were conducted using an online platform (Zoom^R^, Zoom Video Communications). The NAs participated from their workplace. During the fourth FGD participants' comments tended to be redundant from data already collected, thus saturation was assumed (Saunders et al., 2018). All FGDs were audio recorded and transcribed verbatim.

BOX 1 **Interview guide for the Focus Group Discussions**

Focus areas during the discussions

Experiences of work in general during the COVID‐19 pandemic:

- How have you, as staff, experienced the pandemic?
  - How are things today (November 2020) compared with the beginning of the pandemic?
- What has functioned well/less well during the pandemic?
- Is there anything you wish you could have done differently?
- To your knowledge, how have the residents and their relatives experienced the pandemic?

Support and resources during the COVID‐19 pandemic:

- What kind of support have you received at your workplace?
- Have you lacked any support? If so, what support would you have liked?
- Did you receive any specific training during the pandemic?

End of session

- What are the most important learnings to take with you to the next wave/pandemic?
- How has it been to participate in this discussion?
- Is there anything further you would like to add?

### 2.4 Data analysis

Focus group discussions and the initial stages of analysis were conducted in Swedish. The report was produced in Swedish and then translated by the authors to English. All quotes were translated from Swedish to English by the native English speaking author. Data were coded and analysed using Microsoft Word to organise the data.

**FIGURE 1:**
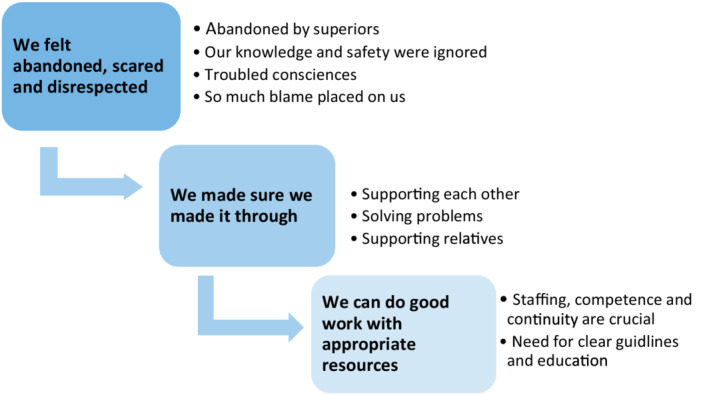
Three themes with sub‐themes identified in the empirical analysis

A descriptive analysis was undertaken in which the transcribed FGDs were analysed using Braun and Clarke's (2006) six‐step thematic analysis methodology. Initially, two researchers independently read each transcript to get a good grasp of the whole material. Data were organised by coding, a process of dividing the text into meaningful units and giving each unit a name or code. Researchers used an inductive approach in which data were coded without trying to fit it into a pre‐existing structure or into the researchers' preconceptions (Braun & Clarke, 2006). The coding process continued until all data was exhausted. Then, codes with similar content were grouped into potential sub‐themes. Examples of potential sub‐themes were ‘fear of being contaminated by COVID‐19’, ‘witch hunt in the media’ and ‘shortage of staff’. During the next step, all potential sub‐themes were reviewed to find related patterns within and between each sub‐theme. We merged sub‐themes considered to have a common origin. When opinions differed between researchers, we went back and reread the transcripts. After discussion, researchers agreed on nine sub‐themes. These were organised into three major themes by relevance, similarities and relationships within content. All authors were involved in the process of refining, framing and naming the themes according to the related sub‐themes (Figure 1). The report was produced, using quotes selected to illustrate each sub‐theme.

### 2.5 Rigour and trustworthiness

We used the concepts of credibility, dependability, confirmability and transferability to maintain rigour in the research process (Guba & Lincoln, 1989). To ensure credibility, the research team systematically followed the guidelines of Braun and Clarke (2006). The initial analysis was performed by two of the authors and then discussed and validated by all authors. Transcripts were not returned to participants for checking, but after each FGD, the discussion was summarised by the moderator to check if the participants agreed on the content or wanted to make adjustments. To increase dependability, we used the same moderator for all FGDs, and the moderator tried to prevent censoring or domination from individual participants and encourage every NAs to speak freely. Throughout the research process, the authors strived to document all steps to ensure transparency. To improve confirmability, we checked to confirm that codes and sub‐themes were grounded in data and that no data were overlooked using reciprocal reading between themes, subthemes and the entire data set. When research members disagreed, we returned to the transcripts and discussed until an agreement was reached. Our research group is comprised of individuals with different backgrounds and perspectives who went through and interpreted data and who could complement and question each other. In addition, to ensure confirmability, quotes are used to illustrate that analysis and findings are grounded in the data. Finally, we attempted to describe the context and sample in a way that allows the reader to judge the transferability of the findings.

### 2.6 Ethical considerations

The study was approved by the Ethical Review Authority in Stockholm, Sweden and confirms to the Declaration of Helsinki (World Medical Association, 2013). All NAs gave written informed consent after receiving written and verbal information about the aim of the study from the researchers. NA identities were removed from transcripts to guarantee confidentiality. Participation was voluntary, and the participants could withdraw at any time without consequences.

## 3 FINDINGS

Three major themes and nine sub‐themes were generated (Figure 1). Quotations illustrate each theme.

### 3.1 We felt abandoned, scared and disrespected

Nursing assistants from all FGDs except one (FGD 4) felt abandoned and ignored by managers and RNs but all NAs felt accused of having spread COVID‐19 to NHs and of providing low quality care by mass media and residents´ relatives. Furthermore, they did not have access to the same high standard of personal protective equipment (PPEs including face mask, vizier and protective clothing) as their colleagues at the hospitals. Hence, they had negative feelings of stress, fear of being infected and infecting others and were conscience‐stricken by not being able to provide high quality care.

#### 3.1.1 Abandoned by superiors

Without adequate knowledge about virus transmission, NAs felt they were forced to handle the situation themselves. They expressed strong feelings of being abandoned without directives and often without support from managers, RNs, or coordinating municipality directors. NAs from three of four NHs recount the physical absence of unit managers, who partly worked from home during the initial spread of infection. It was especially difficult to come in contact with a manager during weekends.

What I remember from that weekend was also that there was some kind of chief on standby that we could contact and who we tried to call, but we did not get in touch with anyone that way either which made it even more stressful because we did not really know what to do or how to do it ourselves (FGD1).

When managers were on site, communication usually occurred only by quick exchanges in the corridor. NAs wanted a present and supportive manager for difficult decisions about how to provide optimal care for a resident or how to best reduce spread of infection. Absence of governance and management led NAs to feel abandoned and alone, not knowing what to do. Even RNs were absent from clinical work in some NHs. NAs perceived that some RNs were afraid of infection and did not dare to enter the ward.

The nurses were often away and some of them did not want to come for a long time. They did not want to come into the ward (FGD 3).

Nursing assistants felt insecure due to lack of information about virus transmission and guidelines for safe management of residents. Furthermore, without RNs present at the NHs, NAs had to act outside their scope of practice in order to meet residents' medical needs. This caused feelings of uncertainty about patient safety.

I really wonder who is in charge, they just abandoned us. They said the nurses are here and you can call them. Of all times I worked with X (a colleague), there was only one time the nurse came to the floor. The resident who had COVID‐19 had insulin that needed to be injected, and we are not allowed to give insulin, but I remember so well that it was X who gave insulin (FGD 2).

Some NAs were afraid of being infected or of transmitting the virus to residents or their own family. As a consequence, they were reluctant to visit infected residents and did not want to meet colleagues who had been exposed to the virus.

Everything changed, I have never seen anything like it in my whole life. The fear in people's eyes was so obvious, everyone was afraid of each other, the psychological trauma was enormous even as the physical work lessened (FGD 2).

#### 3.1.2 Our knowledge and safety were ignored

Nursing assistants felt directly devalued by leaders, first because they felt their safety was not prioritised and then because their knowledge from the pandemic was not appreciated. At the beginning of the pandemic, NAs felt they received lower quality PPEs compared with RNs at the same NH, to staff who came to test the residents for COVID‐19 and to healthcare staff at hospitals. When NAs had to use equipment of lower standard than their colleagues, they perceived that they were not as important and not as valuable as their colleagues. NAs doubted that their PPEs were fit for purpose or as effective as the equipment used in hospitals, but when they asked their managers, they were told that their equipment had been developed for NHs specifically.

There was a special ambulance that drove around everywhere to people who were infected (for testing). They had these incredible space suits and we had nothing. And the nurses had those safe viziers compared to us nursing assistants, so we had to go in with those viziers made from overhead paper that we had to reuse all the time. And they were totally fogged up you could not see anything; you could not care for the residents after a while. But that's what we had, and we were expected to use them several days in a row (FGD 3).

Despite reporting their experiences to RNs and managers, NAs neither received feedback nor observed organisational changes. They were simply told to ‘look ahead’. NAs declare that change must take place for them to be able to provide safe care for residents and protect themselves in case of a new pandemic wave.

The managers came and asked how can we prevent spread of COVID‐19? And I told them everything we have gone through, so they knew. But how they react and use that information, we do not know that. I have not seen any changes on my ward (FGD 2).

#### 3.1.3 Troubled consciences

Staff shortages, increased care needs, time‐consuming use of PPE and frequent telephone calls from worried relatives made it difficult for NAs to provide residents the support and care they needed. NAs felt they were forced to give low priority to personal care such as talking and spending time with residents. This led to loneliness for residents and left NAs insufficient and conscience‐stricken.

They were so alone in their rooms, we are used of being with them all the time and having them around us. I know one colleague brought a TV from home just so they would have something to do. They got what they needed but it was just the loneliness. We tried with newspapers and talked with them when we were in there, but you know you have all that equipment so they could not see it was us either (FGD 4).

Even though NAs knew what the best of care for the residents, they felt they were unable to act according to their professional judgement due to the situation. This led them to feel powerless and sad.

We took care of the un‐infected first and so the others had to wait before they could have something to eat … and it could take a very long time. I know one day we were finished with the morning's work at twelve‐thirty when lunch should be served and the last one had just gotten their breakfast. So, the night fast was extremely long (FGD 3).

Care for residents in palliative situations was even more complex. Residents had to be left alone to a greater extent than usual. When residents passed away, NAs described troubled consciences because they felt they had not been able to provide the best care for the residents.

Many of them have died now. The feeling is hard to describe. After we prepared the bodies and made them ready to leave, we sat on the floor and cried because we needed support that we never got, I will never forget that you go home and feel so awful, and you think ‘did I do everything right today, did I miss something?’ I still have not processed it (FGD 2).

#### 3.1.4 So much blame placed on us

Nursing assistants felt accused of having brought the virus into the NHs and of incompetency in handling COVID‐19 both by mass media and residents' relatives. NAs felt unfairly treated and emphasised that they have the same training as NAs at hospitals and that they have done everything to protect the residents.

There was somewhat of a witch hunt on care workers in nursing homes, that we were the ones who brought the virus into the residences and that it was the older people we were supposed to protect first and foremost. I thought that was really awful last spring. That it was our fault (FGD 4).

### 3.2 We made sure we made it through

Despite feeling abandoned, NAs found ways to manage the situation in the NHs. NAs reported establishing new routines and strategies to solve problems and support residents' relatives. Above all, they supported each other.

#### 3.2.1 Supporting each other

By supporting and helping each other, NAs managed the situation when it was at its hardest both for themselves and the residents. Few NAs were offered professional debriefing or other organised support during the pandemic. On the contrary, they experienced great support from each other in the team, both professionally and privately.

There were five of us who were clearly infected, and we called each other and told, I cannot breathe for example and then the other would call back and say do like this (FGD 2).

#### 3.2.2 Solving problems

Nursing assistants created their own routines, learning from each other and mass media to reduce spread of the virus. When NAs did not receive guidance or support from managers, they had to find their own strategies to cope with the daily work and find ways to avoid spread of infection. An important issue in the beginning of the pandemic was insufficient knowledge about viral transmission. They had seen on TV that healthcare professionals working with COVID‐19 patients in hospitals protected their hair. NAs had no such protective material, so instead they took a shower after each contact with infected residents and before leaving the NH.

So, very often we were left to ourselves. When I worked with X (colleague), we made a decision. We took those with corona first in the morning and then we went to shower and change. One day, we showered eight times. We sanitised everything we needed to use and then we went out to shower and change our clothes, so that we could come back and go to another resident. Regularly, but we did not get tips or advice from anyone. And back then we did not have any protection for our heads, so we used the aprons to cover our heads (FGD 2).

#### 3.2.3 Supporting relatives

Nursing assistants developed novel strategies to support residents' relatives. In Spring 2020, NHs did not allow visitors. Relatives phoned frequently about residents' health status. NAs had understanding for relatives´ concerns but this was time consuming and interrupted their work. Therefore, some NAs decided to schedule for regular phone calls with relatives.

We do not have time, you cannot answer and hold the phone when you have so many clothes that might be contagious and you have to go and shower. That's why I talked to all the relatives and told them that if we do not answer the phone, it does not mean we are ignoring them and that we will call when we can because we have so much clothing and protective equipment (FGD 2).

Nursing assistants arranged for relatives to take a final goodbye to their loved ones for example by moving the bed to a window.

We had the possibility to use the balcony so we could prepare them nicely like we usually do. And then you could move the bed to the balcony and the relatives could come that way. I thought that felt good. Because otherwise it was a little—ok now she died and then she was sent away in that awful sack (FGD 4).

Finding ways to support relatives could be demanding for NAs. Despite the ban, relatives were allowed to visit residents in palliative care with expected short survival time. Some relatives were afraid of infection and did not dare enter the NH. Instead, they asked for digital meetings with the resident. One NA described that she felt uncomfortable partly because she could not ask the resident for consent to a digital meeting but also because she felt relatives were demanding.

Some were terrified. I had those who did not dare set a foot in here, so I had to Skype with them on the deathbed and that felt strange I have to say. I mean it was not like you could get consent from the resident … it felt a little strange to even film him. But the relatives were so happy they could choose the prayer music and that kind of thing. But then they got very demanding, they even wanted me to kiss their father on the forehead. There is a line somewhere (FGD 3).

### 3.3 We can do good work with appropriate resources

Nursing assistants believed it would be possible to fulfil their professional duties even in a pandemic if they had adequate continuous staffing with the right level of competency, as well as guidance and support from robust leaders. By the time of FGDs (9 months into the pandemic), staff members knew how to perform cohort care but point out that adequate staff is a prerequisite for cohort care.

#### 3.3.1 Staffing, competence and continuity are crucial

The condition of staffing before the pandemic were strained in three out of four NHs, leaving staff vulnerable and less able to provide safe care and minimise the spread of COVID‐19. In one NH the NAs were willing to work overtime to avoid external staff with inadequate training who were unfamiliar with the routines and lacked resident knowledge. This NH had no staffing problem and thus cohort care could be provided.

Everyone pulled together … even though many in the staff worked overtime. Just so that we did not need to take in temporary employees and staff from other wards (FGD 4).

In the three other NHs, NAs reported severe staff shortage and many temporary employees, but no recruitment of additional staff. NAs considered NHs to be understaffed and vulnerable with no margins when the pandemic started. When regular NAs were quarantined due to established or suspected infection, staffing became untenable. Consequently, they were too few to maintain cohort care or to isolate residents, especially those with dementia who required extra resources to stay in their rooms.

Cohort care … having enough staff to have someone who only go into the contagious cases, we did not have enough people for that. Our basic staffing is such that we have two in the morning and two in the evening so that was already not going to happen. It is not easy to keep people with dementia isolated in their rooms (FGD 3).

Nursing assistants highlighted how crucial continuity of staff was for maintaining safe and high‐quality care. Furthermore, the NAs emphasised that knowledge and education are factors that greatly affect the quality of care normally and especially during the pandemic. They had experience from working with temporary staff with limited competence in hygiene routines and with scarce knowledge about how to care for persons with dementia.

I understand the economy is haemorrhaging but the staff is so important, that you know Swedish. And that this is not a playground, these are people we are taking care of. And it is important with basic hygiene and how we handle it, it is so extremely important. I said (to a temporary worker); ‘you need to wash your hands, you need to disinfect your hands’, and I just get nonchalance back like … I do not need to listen to you. So, the staff is very important (FGD 2).

#### 3.3.2 Need for clear guidelines and education

Nursing assistants experienced limited guidance on COVID‐19 prevention and control at the beginning of the pandemic. Guidance and regulations changed frequently, sometimes daily and were conflicting and confusing.

If you were gone one day you had new routines, and they were very different (FGD 4).

At one NH, NAs received training in hygiene routines and how to use PPEs as early as March 2020. At the other NHs, NAs felt they had too little knowledge about hygiene routines and virus transmission. NAs expressed a need for specific training and proposed introduction of a designated colleague to monitor implementation of guidelines and to answer questions about hygiene, aseptic techniques and routes for transmission of the virus.

We had this piece of paper where it said how we should take off and on protective gear, and we hung it up on the wall. Yeah, it was not so straightforward, so we felt pretty unsure about how and what we should do (FGD 1).

Nine months into the pandemic, many NAs expressed that they had learned a lot and had clearer and more well‐established routines for if or when the virus returns.

## 4 DISCUSSION

The study objective was to illuminate the experiences from Swedish NAs working in NHs during the first wave of the COVID‐19 pandemic. Their experiences are reflected in three main themes: (a) We felt abandoned, scared and disrespected (b) We made sure we made it through and (c) We can do good work with appropriate resources (Figure 1). A key finding was that the NAs felt abandoned and left alone with a huge responsibility for the care of the residents, especially in case of absence of the RNs.

Inconsistent guidelines, lack of integrated teamwork and the absence of leadership during the pandemic has led to heavy work overload for NAs with responsibility for the day‐to‐day care of the older people. NAs experienced limited and contradictory guidelines, especially regarding hygiene routines, and therefore developed their own strategies. One possible explanation for contradictory information may be fragmented NH organisation, where the municipality and the region share responsibility for NHs. Healthcare professionals working in NHs and at other long‐term care settings in the United States found directions from local, state and federal agencies not only confusing but also contradictory, as in this study (White et al., 2021).

Nursing assistants in three of four NHs experienced lack of integrated leadership and reported how team collaboration across professions diminished and even disappeared. This forced NAs to carry a great burden alone while other team members were at a distance. Our results are in line with a recent Swedish study showing that NAs in home care felt abandoned by first line managers who escaped the risk of infection by staying in secure offices (Rücker et al., 2021). It is remarkable that NAs were left alone, because quality care for frail older people depends on a trustworthy team including NAs, RNs managers and physicians (Eldh et al., 2016; Lundgren et al., 2016; Szebehely, 2020). Earlier research has shown that visible, active and robust leadership is essential to cope with an extraordinary situation such as a pandemic, since leadership is often a determining factor for staff's resilience to high workload and stress (Digby et al., 2021; Losty & Bailey, 2021; Lundgren et al., 2016; Rücker et al., 2021). Exemplary leadership during uncertain circumstances such as in a pandemic could be RNs acting as role models, working alongside frontline staff (Shanafelt et al., 2020; Yau et al., 2021). In the current study, NAs describe positive experiences only in cooperation with their NA colleagues, and no sense of team spirit with RNs, managers or physicians. Contrary to our study, others have been able to show that team spirit in some health care settings increased during the pandemic (Billings et al., 2021; Rücker et al., 2021).

This study has not explored reasons for RNs and first line managers absence in NHs. However, according to local and national evaluations in Sweden, there seems to be various reasons for this absence. In view of RN shortage, they may have preferred not to be exposed to residents in order to reduce residents´ and their own risk for transmission of COVID‐19 (The Health and Social Care Inspectorate, 2021). Reports have even showed how RNs in some cases received directives from superiors to refrain from visiting residents with infection (The Health and Social Care Inspectorate, 2021). These guidelines on physical distance have been perceived and interpreted differently in different units (Boström et al., 2020; The Health and Social Care Inspectorate, 2021). The health care authority issued guidelines recommending physicians avoid unnecessary visits at the NHs with the aim to limit virus transmission (Boström et al., 2020; The Health and Social Care Inspectorate, 2021). These guidelines may have been interpreted to mean that even RNs should avoid unnecessary visits in NHs. Many NH residents with covid symptoms were not assessed by a physician or a RN (The Health and Social Care Inspectorate, 2021). Furthermore, in the beginning of the pandemic in Sweden, there was a PPE shortage which could have led to reduced contacts with infected residents to save on PPE (Kabir et al., 2020).

Despite abandonment, lack of leadership and contradictory guidelines, NAs in our study were able to pool their resources and use their previous knowledge to solve day‐to‐day problems during the pandemic. They developed strategies to cope with their own worries and fears and at the same time provided the best possible care. It is unacceptable that not all NAs were offered professional debriefing or other organised support as recommended (Stafford et al., 2021), so they were left to support each other. NAs felt that they were forced to give low priority to personal care such as talking and spending time with residents. This led to loneliness for residents and left NAs feeling insufficient. The high number of deaths during a short time frame and under these special circumstances provoked many negative feelings among NAs, which can be summarised as moral distress. Moral distress occurs when NAs are unable to act according to their own professional judgement and/or personal values due to external constraints or internal characteristics (Deschenes et al., 2020). Also, challenges resulting from many patients dying during a short and special period may lead to moral distress especially when patients are dying in absence of their family and when traditional end‐of‐life practices are bypassed to protect staff from exposure (Deschenes et al., 2020; Kelley et al., 2021). Likewise, others have reported a high degree of moral distress among nurses, especially women working as frontline healthcare providers during the pandemic (Lai et al., 2020). NAs in current study felt that no one was listening to their experiences and views. They wanted to contribute to the development of sustainable working routines for the pandemic. This is in line with findings from former studies reporting that NAs feel undervalued, powerless, marginalised, and excluded from involvement in care decisions (Cronin et al., 2020; Güney et al., 2021).

Nursing assistants spend more time with residents in NHs than any other group of health workers, providing both physical and emotional care and are an important team member. Despite the critical role of NAs, they are often invisible in the planning and organisation of care for residents. There may be many explanations for this but one critical issue for NAs in NHs in Sweden, as in many other countries, are predominantly under educated, poorly paid, women and a high proportion born outside Sweden which contributes to low status of NAs. In 2018, 29 percent of the NAs in home care, home health care and nursing homes were born outside Sweden (Statistics Sweden, 2020). In large cities as Stockholm, the foreign‐born staff in some units is the dominant staff category. In other parts of the world, there are similar patterns in older people care (Hurtado et al., 2012; Simmons et al., 2021).

A societal crisis calls for national leadership as well as a robust local leadership in NHs. In the light of current knowledge, political efforts are necessary to improve the care for older people in Sweden. Some of the most important factors are to strengthen competence by giving both new and existing staff the opportunity for education and training, as well as ensuring that adequate medical competence is available in NHs.

### 4.1 Strengths and limitations

Focus group discussions are suitable to help participants to explore and clarify their experiences, but differences in group dynamics, size of the groups and familiarisation of the participants raise ethical issues (Dahlgren et al., 2007). Each FGD had between three and seven participants because smaller groups are more suitable when participants are deeply involved in the topic (Dahlgren et al., 2007). Due to social distancing recommendations, we used an online platform (Zoom) to collect data. This could affect group dynamics as it might be difficult for the moderator and the observer to facilitate discussion. However, participants from each NH were assembled in a room adjacent to the NH with a computer with a camera in front of them and the moderator and observer facilitating the discussion at distance. The researchers experienced that the participants interacted and discussed the research questions in the group freely which could have depended on that the participants knew each other and therefore dared to speak openly about difficulties.

We experienced that it was difficult to recruit NHs to participate in FGD because of a turbulent period with shortage of staff. Therefore, we involved managers of NHs to ask interested NAs to participate. In this case, there is a power differential between managers and NAs with the risk that NAs felt forced to participate and/or be afraid to talk about negative experiences. Consequently, there is a possibility we did not manage to capture the full range of NAs negative experiences. Men working as NAs in NHs are underrepresented and it is possible that a FGD with additional male respondents have influenced the result in a different way.

The qualitative approach that was used is valuable for providing rich descriptions of complex phenomena. This study reflects views of skilled and experienced NAs who have had permanent positions for a long time from a specific region of Sweden (Table S1). Other views may be held by temporary staff with less education and experience which is common in many NHs in Sweden (Szebehely, 2020), this may consequently restrict the transferability of the findings. Furthermore, NAs from FGD 4 reported that they usually had managers and RNs present in the NHs which may have led to another arrangement of staffing density. Despite, the presence of superiors they felt increase stress at work due to the exceptional situation with lack of adequate PPE and pressure from media and relatives. However, this qualitative study is too small to draw any conclusions about how the presence of superiors affected the NAs experiences in large.

## 5 CONCLUSIONS AND IMPLICATION FOR PRACTICE

Nursing assistants worked under evolving, stressful circumstances during the first wave of the COVID‐19 pandemic, and they reported feeling abandoned and burdened. Significant steps need to be taken to improve or maintain the wellbeing of NAs and to ensure that patient safety is sustainable, during the ongoing COVID‐19 pandemic and beyond. NAs have an essential role in providing care for vulnerable older people and ought to be integrated into interprofessional teams with robust leadership especially during crisis, to ensure safe and high‐quality care. Furthermore, NAs need the support of clear and structured guidelines to feel safe and perform their work according to best practice. NH managers may find our findings useful to ensure that NHs will stand prepared and equipped for future crises. Finally, to provide a more sustainable working environment for NAs in NHs, they need opportunities to reflect over their working conditions and experiences, as well as be offered a combination of peer, organisational and professional support.

This study raises future research questions about how NAs knowledge, education and work satisfaction affect residents' health and well‐being. The views and experiences of NAs should be considered in future research and for development of NH care and routines.

## FUNDING INFORMATION

Health Research Fund, Region Stockholm (ALF grant nr 20200202), Research support for Network Healthcare, Region Stockholm (grant nr FoUI‐937161), Swedish order of Freemasons, Research Fund for Rehabilitation and Medical Research and Funds from Foundations at Karolinska Institutet (grant nr 2020‐01839).

## CONFLICT OF INTEREST

None of the authors have a conflict of interest to disclose.

## Supporting information


**Appendix S1** Supporting Information

## Data Availability

The data that support the findings of this study are not publicly available due to ethical restrictions.
