# Supplementary material for: How much are we worth? Experiences of nursing assistants in Swedish nursing homes during the first wave of COVID‐19
Source: Int J Older People Nurs. 2022 Aug 17:e12498. Online ahead of print. doi: 10.1111/opn.12498 (PMC9539304; doi:10.1111/opn.12498)
Supplement: Supplementary file 1 — Appendix S1 Supporting Information [file OPN-9999-0-s001.docx]

**Supplement 1. Nursing homes and nursing assistants’ characteristics**

Nursing homes in Region Stockholm (n=318)

In May 2020, Region Stockholm had 318 nursing homes (NHs) with a median of 73 (IQR 42; 107, min-max 1-377) residents.

Socioeconomic characteristics of NHs

We used Care Need Index CNI (Sundquist et al., 2003), a social deprivation index, in order to describe the socioeconomic location of the NHs.

CNI includes several dimensions: relative percentage of elderly persons living alone, children under age 5, unemployed people, people with low educational status, single parents, high mobility, and foreign-born people.

Statistics Sweden (SCB, 2021) provides CNI for each municipality. A higher value indicates lower socioeconomic status. For the 26 municipalities in Region Stockholm, CNI ranges from 0,65 (highest socioeconomic status) to 2,3 (lowest socioeconomic status), with a median of 1,1 (IQR 0,9; 1,3).

Mortality rates per NH during early COVID-19 pandemic year 2020

Mortality rates among the 318 NHs were extracted from VAL database and calculated as “mean number of deaths per month during the 3-month period March-June 2020 per number of residents per NH” and ranged from 0 to 9%. VAL database is the administrative health data register of region Stockholm (Vårdanalysdatabasen, VAL; Stockholm regional healthcare data warehouse).

Nursing homes recruited for participation

We approached NHs according to their size allowing nursing assistants (NA) to participate in a Focus Group Discussion (*n* =82). We recruited four NHs differing regarding their size, socio economy and mortality rates (see Table 1).

Physical presence of RNs and managers differed between NHs during the first wave of the pandemic. FGD 4 reported that the RNs and managers were present most of the time at their NH but all other three FGDs reported that they were not present at all. Staff availability also differed between NHs. FGD 4 reported no problems with staffing as NAs themselves worked overtime when needed, and their manager was able to quickly recruit additional staff. Furthermore, one NH (FGD 1) was not affected as heavily by the COVID-19 pandemic as the others and only had a few infected residents.

**sTable1. Characteristics of recruited nursing homes (NH) (n=4) and nursing assistants (NA) (n =20)**

| **NH** | **Size of NH^1^** | **Socio-economy^2^** | **Mortality rate^3^** | **Part of Stockholm** | **NO NA** | **Age of NAs** | **Years at current NH** |
| --- | --- | --- | --- | --- | --- | --- | --- |
| FGD 1 | Medium | Low | 2% | South | 5 | 31-40  41-50  51-60  Unknown  Unknown | < 1  > 5  > 5  Unknown  Unknown |
| FGD 2 | Large | High | 5% | North | 5 | 25-30  41-50  51-60  61-65  Unknown | > 5  1-3  < 1  > 5  Unknown |
| FGD 3 | Large | Low | 5% | South | 7 | 31-40  51-60  51-60  51-60  51-60  51-60  >65 | 3-5  > 5  > 5  > 5  > 5  > 5  > 5 |
| FGD 4 | Small | Low | 1% | South | 3 | <25  51-60  51-60 | > 5  > 5  > 5 |

^1^ Small = 50 or fewer residents, medium = 50-100 residents or large =100 or more residents.

^2^Low: CNI > 1,3; high CNI < 0,9.

^3^ Mean number of deaths per month during the 3-month period March-June 2020 per number of residents per NH.

**References**

Sundquist, K., Malmstrom, M., Johansson SE., & Sundquist, J. (2003). Care Need Index, a useful tool for the distribution of primary health care resources. *J Epidemiol Community Health,* 57(5):347-52.

Statistics Sweden [SCB], Care Need Index (CNI), Retrieved May 19, 2021 from: https://www.scb.se/vara-tjanster/bestall-data-och-statistik/regionala-statistikprodukter/care-need-index-cni/
